# Vitamin K2 Supplementation Reduces Cardiometabolic Risk Factors in Young Adults with Overweight and Obesity—A Randomized Placebo-Controlled Trial

**DOI:** 10.3390/biomedicines14051011

**Published:** 2026-04-29

**Authors:** Xochitl Citlalli Olivares-Ochoa, Iris Monserrat Llamas-Covarrubias, Sergio Sánchez-Enríquez, Andres López-Quintero, Yahatziri Salinas-Varela, Miriam Partida-Pérez, Monserrat Macías-Carballo, Edgar Alfonso Rivera-Leon

**Affiliations:** 1 Instituto de Nutrigenética y Nutrigenómica Traslacional, Centro Universitario de Ciencias de la Salud, Universidad de Guadalajara, Guadalajara 44340, Mexico; xochitlolivaresochoa@gmail.com (X.C.O.-O.); iris.llamas@academicos.udg.mx (I.M.L.-C.); andres.lopezq@academicos.udg.mx (A.L.-Q.); patricia.salinas3709@alumnos.udg.mx (Y.S.-V.); 2 Departamento de Biología Molecular y Genómica, Centro Universitario de Ciencias de la Salud, Universidad de Guadalajara, Guadalajara 44340, Mexico; 3 División de Ciencias Biomédicas, Centro Universitario de Los Altos, Universidad de Guadalajara, Tepatitlán de Morelos 47620, Mexico; sergio.enriquez@cualtos.udg.mx (S.S.-E.); monserrat.macias@cualtos.udg.mx (M.M.-C.); 4 Programa de Doctorado en Genética Humana, Centro Universitario de Ciencias de la Salud, Universidad de Guadalajara, Guadalajara 44340, Mexico; 5 Departamento de Ciencias Médicas, Centro Universitario de Los Altos, Universidad de Guadalajara, Tepatitlán de Morelos 47620, Mexico; miriam.partida@academicos.udg.mx

**Keywords:** vitamin K, dietary supplements, cardiometabolic risk factors, young adult

## Abstract

**Background/Objectives**: Obesity in young adults is a major public health concern and a key contributor to cardiometabolic risk. Vitamin K2 (VK2) has been proposed as a potential adjuvant therapy; however, evidence from randomized controlled trials remains limited. This study evaluated the effect of VK2 supplementation on cardiometabolic risk factors in young adults with overweight or obesity. **Methods**: In this 12-week randomized, double-blind, placebo-controlled trial (NCT05995522), men and women aged 18–35 years with overweight or obesity (BMI 25–40 kg/m^2^) were assigned to receive VK2 (menaquinone-4, 100 µg/day) or placebo. Both groups received standardized nutritional counseling. Body composition, blood pressure, glucose homeostasis, lipid profile, and vitamin K-dependent proteins were assessed at baseline and post-intervention. Between-group differences were analyzed using ANCOVA adjusted for baseline values. **Results**: Forty-six participants completed the study (placebo *n* = 24; VK2 *n* = 22). VK2 supplementation significantly reduced total cholesterol (−10.64 mg/dL, *p* = 0.038) and LDL cholesterol (−6.12 mg/dL, *p* = 0.005) compared with placebo. A reduction in systolic blood pressure showed a trend toward significance (−5.56 mm Hg, *p* = 0.067). No significant effects were observed on body composition, glucose metabolism, or vitamin K-dependent proteins. **Conclusions**: VK2 supplementation resulted in improvements in total and LDL cholesterol levels, with no significant changes in vitamin K-dependent proteins, and may represent a safe and potentially beneficial adjunct to nutritional strategies aimed at early cardiometabolic risk modulation.

## 1. Introduction

Worldwide, obesity is recognized as one of the most significant public health challenges, affecting children, adolescents, and adults alike [1]. Obesity is a chronic condition characterized by excessive accumulation of adipose tissue and is considered a multifactorial disease involving complex and bidirectional metabolic, biomechanical, and psychosocial interactions [2,3]. Over the past four decades, the prevalence of obesity among young adults has increased substantially, rising from 5.5% to 32.6% [4].

During young adulthood, many individuals experience significant changes in lifestyle habits that influence their dietary patterns, recreational activities, and levels of physical activity [5,6,7,8]. Prolonged exposure to unhealthy behaviors—such as poor diet and sedentary lifestyle—combined with environmental and genetic factors during this critical period, may substantially increase the risk of developing obesity [9,10,11]. It is a major contributor to cardiometabolic risk, as adipose tissue dysfunction promotes pathophysiological mechanisms including insulin resistance (IR), dyslipidemia, and systemic inflammation [12,13].

Cardiometabolic risk has been defined as a co-occurrence of metabolic and cardiovascular conditions that encompass abdominal obesity, IR, elevated blood pressure (BP), and dyslipidemia. This cluster of factors elevates the risk of subsequent cardiovascular disease (CVD) and type 2 diabetes (T2D) [14,15]. Individuals with obesity have a higher prevalence and a 2- to 8-fold increased risk of cardiometabolic disturbances compared to healthy individuals [16].

Obesity management requires a multifaceted approach due to its complex etiology [17]. Traditional strategies for the treatment of obesity and the reduction of cardiometabolic risk factors include lifestyle modifications, dietary changes, and increased physical activity [18,19,20]. Additional treatment options include anti-obesity medications, very low-calorie diets, and bariatric surgery for severe cases [17,18]. Although energy-restricted diets are effective tools for improving body weight and cardiometabolic health, their clinical application is limited by poor long-term adherence [21].

Therefore, supplementation with specific nutrients has been investigated as an adjuvant strategy in the management of obesity, aiming to improve cardiometabolic risk parameters. One such nutrient is vitamin K (VK), a group of fat-soluble compounds sharing a bicyclic quinone core and variable side chains that define its isoforms [22,23].

The two main natural forms are vitamin K1 (VK1) or phylloquinone and vitamin K2 (VK2) or menaquinone (MK) [22]. VK1, predominantly obtained from green leafy vegetables, contains a branched aliphatic side chain with a double bond and is primarily associated with hepatic functions, particularly blood coagulation. Its relatively short half-life of approximately 24 h allows efficient storage and utilization within the liver [23,24,25].

VK2, in contrast, comprises several homologs mainly produced through bacterial synthesis, which explains its presence in fermented foods and animal-derived products such as meat and dairy, while also permitting limited endogenous production. The isoprenoid side chain of VK2 varies in length, giving rise to homologs designated MK-2 to MK-15 [23,24,25]. With a longer half-life of up to 72 h, VK2 circulates for extended periods and acts on extrahepatic tissues, including bone and the vascular system. These properties underline their broader physiological roles, including skeletal metabolism, vascular health, regulation of inflammation and oxidative balance, and tissue mineralization [26].

VK plays an essential role in many physiological processes, functioning as a cofactor for the γ-carboxylation of vitamin K-dependent proteins [27]. During this activity, a post-translational modification of glutamic acid residues occurs, catalyzed by the enzyme γ-glutamyl carboxylase, which converts these residues into γ-carboxyglutamic acid [28]. Certain vitamin K-dependent proteins, including osteocalcin and growth arrest-specific 6 (Gas6), have been suggested to contribute to cardiometabolic regulation [29,30,31,32].

In line with this, VK supplementation has shown promising effects on cardiometabolic risk factors in various models [33,34,35,36,37,38,39,40,41]. However, current evidence remains insufficient to draw definitive conclusions. Therefore, randomized, placebo-controlled trials are warranted to further elucidate the true potential of VK supplementation in the management of cardiometabolic disorders [42]. In this context, the present study aimed to evaluate the effect of VK2 (MK-4) supplementation on cardiometabolic risk factors in overweight or obese young adults.

## 2. Materials and Methods

### 2.1. Study Design

We performed a parallel, randomized, double-blind, placebo-controlled clinical trial. This study was designed and reported according to the Consolidated Standards of Reporting Trials (CONSORT) guidelines [43].

Participants received VK2 (MK-4) at a dose of 100 µg/day (Nutricost, Vineyard, UT, USA) or placebo for 12 weeks, with intake recommended alongside meals containing fat. Both groups received initial nutritional counselling aimed at promoting healthy eating habits. The counselling emphasized balanced plate composition with representation of all food groups, variety in food choices including colorful fruits and vegetables, and reduced consumption of sugar-sweetened beverages [44,45].

### 2.2. Participants and Eligibility Criteria

Eligible volunteers were men and women aged 18 to 35 years, with a body mass index (BMI) between 25 and 40 kg/m^2^ and without known health conditions. Participants were Mexicans and were not taking any medications or supplements, nor following a hypocaloric or low-fat diet at the beginning or during the study and were not pregnant or lactating.

### 2.3. Sample Size

Sample size was calculated using a formula the following for comparing two means:

$$n=\frac{{2\left(Z_{\alpha /2}+Z_{1-\beta }\right)}^{2}{\left(SD\right)}^{2}}{d^{2}}$$

Serum levels of carboxylated osteocalcin (cOC) were considered as the outcome variable based on the study by Aguayo et al., with a 95% confidence level, 80% statistical power, and a significance level of *p* < 0.05 [36]. A minimum of 24 participants per study group was determined, accounting for an anticipated 20% dropout rate.

### 2.4. Randomization and Blinding

The study was conducted at the Translational Nutrigenetics and Nutrigenomics Institute, University Center for Health Sciences, University of Guadalajara, Mexico. Recruitment was carried out through both physical and digital media. Participants were enrolled between September and December 2023. Screening included completion of a medical history questionnaire and anthropometric assessments.

An independent researcher not involved in recruitment, assessment, or intervention delivery generated the allocation sequence using a computer-generated simple randomization procedure in R software version 4.0.3 (R Foundation for Statistical Computing, Vienna, Austria). The allocation sequence was kept by the independent researcher and was not accessible to investigators enrolling participants. Upon enrollment, each participant was assigned a study code corresponding to the pre-generated randomization list, thereby ensuring allocation concealment. The allocation sequence was implemented using coded containers prepared by personnel not involved in participant enrollment or assessment, with study supplements pre-labeled according to the randomization list to maintain concealment throughout the trial.

The trial was conducted under double-blind conditions. Participants, care providers, data collectors, outcome assessors, and data analysts were blinded to group allocation. The intervention and placebo were identical in appearance and taste to ensure blinding. Blinding was maintained throughout the study and was only broken after completion of the statistical analysis, with no instances of unblinding reported during the trial. No patients or members of the public were involved in the design, conduct, or reporting of this study.

### 2.5. Study Assessments and Outcomes

The primary outcome of the study was serum carboxylated osteocalcin, selected based on its role as a vitamin K-dependent protein. Secondary outcomes included cardiometabolic risk factors, such as body composition, blood pressure, glucose homeostasis and blood lipid profile parameters.

#### 2.5.1. Cardiometabolic Risk Factors

All variables were assessed at baseline and at the end of the study by trained personnel following standardized protocols to minimize measurement error and ensure reproducibility. Height and weight were measured to calculate BMI, defined as weight (kg) divided by height squared (m^2^). Height was obtained using a portable mechanical stadiometer (Seca, model 213; Seca GmbH & Co. KG, Hamburg, Germany). Participants were measured barefoot, standing upright with their buttocks against the stadiometer, and their heads aligned with the Frankfort horizontal plane. Body weight was measured using a Tanita TBF-300 bioelectrical impedance scale (Tanita Corporation, Arlington Heights, IL, USA). During the measurement, participants stood barefoot and faced forward. This device was also used to estimate body fat percentage and fat-free mass according to the manufacturer’s instructions.

Waist circumference was measured with a Lufkin metallic tape measure (Lufkin, Grand Prairie, TX, USA) at the midpoint between the lower margin of the last rib and the upper border of the iliac crest along the mid-axillary line. Measurements were taken with the participant standing and at the end of a normal expiration. Blood pressure was measured using an automatic aneroid sphygmomanometer (Omron, model HEM-7220 LA; Kyoto, Japan) after participants had rested quietly for 15 min. Measurements were taken on the left arm, in a seated position, with feet flat on the floor and uncrossed, the back supported, and the arm relaxed and resting on a level surface at heart height.

For the assessment of blood lipid profile and glucose homeostasis, venous blood samples were collected in the morning after an overnight fast of at least eight hours. Samples were drawn from peripheral forearm veins using yellow-top BD Vacutainer^®^ tubes containing a clot activator and gel separator (BD, Franklin Lakes, NJ, USA). Blood samples were centrifuged at 3000 rpm for 15 min at room temperature to obtain serum. Serum concentrations of total cholesterol, HDL cholesterol, triglycerides, and glucose were determined using the VITROS^®^ 350 System (Ortho Clinical Diagnostics, Raritan, NJ, USA). LDL cholesterol was calculated automatically by the system using Friedewald’s formula: LDL cholesterol = total cholesterol (mg/dL) − HDL cholesterol (mg/dL) − [triglycerides (mg/dL)/5].

Serum insulin concentrations were measured by enzyme-linked immunosorbent assay (ELISA) using the Insulin ELISA kit (DRG International, Inc., Springfield, NJ, USA). Insulin resistance was estimated by the homeostasis model assessment of insulin resistance (HOMA-IR), calculated as: HOMA-IR = [fasting glucose (mg/dL) × fasting insulin (µU/mL)]/405.

#### 2.5.2. Vitamin K-Dependent Proteins

Concentrations of undercarboxylated osteocalcin (ucOC), cOC and Gas6 were measured using ELISA kits from MyBioSource (MyBioSource, San Diego, CA, USA). The ucOC-to-cOC ratio (UCR) was determined as an index reflecting the proportion of circulating ucOC to cOC.

#### 2.5.3. Lifestyle

Food intake was assessed using a 24-h dietary recall for a typical day, with assessments conducted at 4-week intervals throughout the study period and analyzed using Nutritionist Pro Diet software (version 7.9; Axxya Systems, Stafford, TX, USA). Dietary intake of VK2 (MK-4) was estimated based on values from the 2019–2020 Food and Nutrient Database for Dietary Studies (FNDDS) of the United States Department of Agriculture (USDA). As the database does not provide information on other MK, intakes of MK-5 to MK-11 were estimated based on published studies quantifying the content of these compounds in foods [46,47,48,49,50,51,52,53]. Additionally, a validated food frequency questionnaire targeting VK1-rich foods was administered to assess dietary intake over the preceding 3 months and was applied at both baseline and at the end of the study period [54].

Physical activity was evaluated at baseline using the International Physical Activity Questionnaire (IPAQ). Weekly physical activity was quantified using the physical activity index (METs × frequency × duration), and participants were classified into low, moderate, or high activity levels. Tobacco use was also assessed at baseline using a single dichotomous question, with responses coded as “yes” for current smokers and “no” for non-smokers.

#### 2.5.4. Adherence

The count of unused capsules was assessed as an indirect method conducted by an unblinded investigator. All patients provided consent to receive reminders sent to their personal contacts to encourage compliance with the intervention. This procedure was maintained throughout the study in both groups.

#### 2.5.5. Treatment-Related Effects

The presence of gastrointestinal symptoms, including diarrhea, vomiting, nausea, among others, was assessed at baseline to determine whether participants experienced any discomfort prior to the intervention. Symptom severity was evaluated based on frequency, intensity, and duration. Participants were subsequently monitored at each follow-up visit to assess the occurrence of adverse effects associated with supplementation.

#### 2.5.6. Participant Compensation

At the end of the 12-week intervention, participants received a personalized dietary plan aimed at reducing cardiometabolic risk factors [20,44].

#### 2.5.7. Statistical Analysis

The Shapiro–Wilk test was used to assess normality of the data. Categorical variables are presented as frequencies and percentages, whereas continuous variables are reported as mean ± standard deviation or as estimated mean difference (95% CI) for parametric data, and as median and 25th–75th percentiles for non-parametric data. Baseline comparisons between groups were performed using the chi-square test, independent *t*-test, or Mann–Whitney U test.

Changes in food intake variables within groups over time were evaluated using the Wilcoxon signed-rank test for variables assessed at two time points, and the Friedman test for variables with more than two repeated measurements. Between-group differences in all assessed variables were analyzed using analysis of covariance (ANCOVA) with Bonferroni adjustment for multiple comparisons, adjusting for baseline values. Effect size was estimated using eta squared (η^2^) derived from the ANCOVA [55]. Statistical analyses were performed using SPSS^®^ Statistics version 25.0 (IBM Corp., Armonk, NY, USA), and statistical significance was set at *p* < 0.05.

## 3. Results

### 3.1. Baseline Characteristics of the Participants

**Figure 1:**
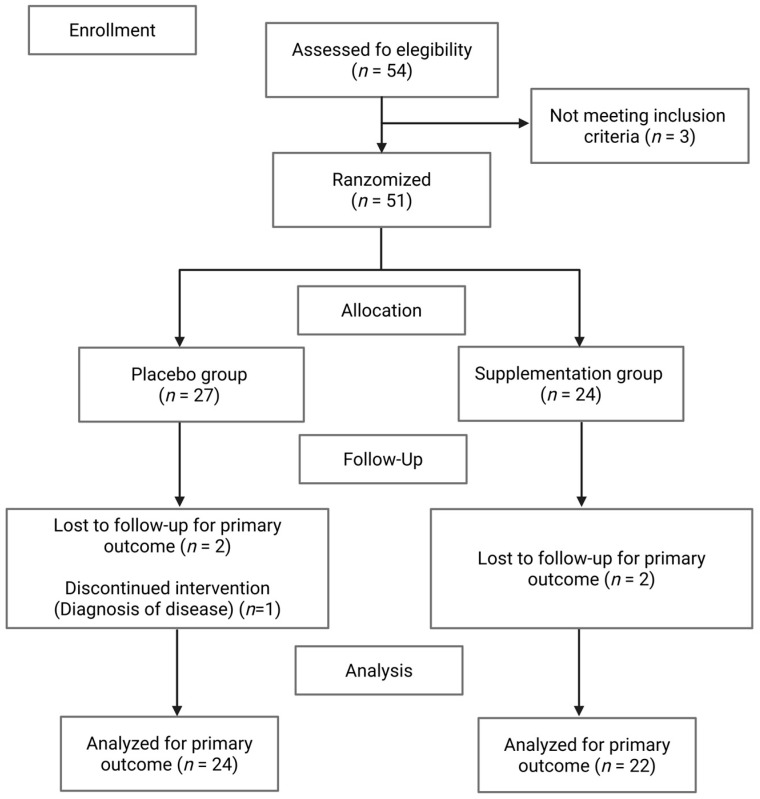
CONSORT flow diagram of participants during the intervention. Of the 54 young adults assessed for eligibility, 51 met the inclusion criteria, and 24 and 22 participants completed the placebo and supplementation groups, respectively.

Fifty-four participants were assessed for eligibility; however, only fifty-one young adults of both sexes with overweight or obesity and no known comorbidities were included in this study; of these, 24 in the placebo group and 22 in the supplementation group completed the intervention (Figure 1).

The population had a mean age of 26.96 ± 4.08 years and a median BMI of 29.9 [27.80–33.04] kg/m^2^, with 41.2% of the participants being female (Table 1). Diastolic BP and serum triglycerides levels were significantly higher in the placebo group compared to the supplementation group at baseline (*p* = 0.019 and *p* = 0.049, respectively).

At baseline, there were no significant differences between the placebo and supplement groups in smoking status or physical activity levels (*p* > 0.05) (Table 2). The median energy intake in the overall sample was 1916.69 [1125.16–4929.73] kcal/day, with comparable macronutrient and micronutrient intake between groups.

### 3.2. Effect of VK2 (MK-4) on Cardiometabolic Risk Factors

After the intervention, no changes were observed in body composition, glucose homeostasis, or vitamin K-dependent proteins (Table 3). A decrease in systolic BP with a trend towards statistical significance (*p* = 0.067) was observed in the supplementation group compared to the placebo group. Within the lipid profile, total cholesterol significantly decreased in the supplementation group compared with the placebo group (*p* = 0.005, η^2^ = 0.096). LDL cholesterol was also significantly lower in the supplementation group than in the placebo group (*p* = 0.038, η^2^ = 0.169).

### 3.3. Changes in Nutrient Intake During the Intervention

Within the assessment of nutrient intake during the intervention, in the placebo group, we observed an increase in sodium intake from baseline to week 4, and from week 4 to 8, followed by a decrease back to baseline levels by the end of the intervention (Table S1). In the supplementation group, VK1 intake decreased after the intervention, and an increase in VK2 intake between baseline and 8 weeks of intervention; however, intake levels returned to baseline by week 12, and total VK intake did not show any significant difference (Table S2). When comparing the changes after the intervention between groups, we observed that most nutrients remained unchanged, except for VK2, which increased in the placebo group; however, VK1 and total VK intake did not differ between the groups (Table 4).

Treatment adherence was 84.17 ± 7.89% in the total population, with no significant differences between study groups (*p* = 0.690). One participant in the supplementation group reported gastrointestinal symptoms shortly after initiating the intervention. These symptoms were continuously monitored by a study physician and resolved within a few days without any intervention. No other concurrent events were observed.

## 4. Discussion

Individuals with a BMI ≥ 25 kg/m^2^ are more likely to present alterations in cardiometabolic risk factors [56]. VK2 has been proposed as a potential modulator of these parameters. Accordingly, previous literature has emphasized the need for randomized, placebo-controlled trials to clarify the therapeutic potential of VK supplementation in the management of cardiometabolic disturbances [42].

No significant changes were observed in body composition variables such as BMI, waist circumference, and body fat percentage after the intervention in this study. Although an inverse relationship has been reported between VK status and parameters such as BMI and fat mass, and VK2 supplementation has been shown to reduce fat accumulation in animal models, studies conducted in healthy adults, individuals with T2D, and postmenopausal women have not shown significant effects on these variables [33,38,39,57,58,59].

Elevated systolic blood pressure (BP) was the leading modifiable risk factor for premature cardiovascular disease (CVD) mortality worldwide in 2021, accounting for an estimated 10.8 million deaths [60]. In this study, we observed a reduction in systolic BP of 5.56 mm Hg, with a trend toward statistical significance (*p* = 0.067). A clinical trial using VK2 (MK-7) supplementation in healthy individuals reported similar findings for diastolic BP, with marginal statistical significance (*p* = 0.05) [37]. In our study, although a reduction in diastolic BP was observed in the supplementation group, this difference was not statistically significant compared to placebo group (*p* = 0.453).

It is important to consider that the placebo group presented higher baseline BP values, which may have influenced the observed effects. Although no statistically significant differences were observed at baseline for systolic BP, the placebo group showed numerically higher values. Over the course of the study, a trend toward reduction in systolic BP was observed in the supplementation group (*p* = 0.067); however, a parallel decrease in the placebo group may have attenuated the apparent between-group effect.

For diastolic BP, baseline values were significantly higher in the placebo group. Notably, the placebo group started with values further from the population mean reported for young Mexican adults (~113.3/70.5 mm Hg), whereas the supplementation group was closer to these reference levels [61]. This difference is relevant, as it may have amplified a regression-to-the-mean effect in the placebo group, leading to reductions over time as values tended to approach the population average. Consequently, decreases observed in the placebo group in both systolic and diastolic BP may have obscured a potential effect of supplementation. Although analyses were adjusted for baseline values, the influence of these initial imbalances cannot be entirely ruled out.

Human studies suggest that VK may play a role in this risk factor. The combination of low vitamin D and VK levels has been associated with increased BP and a higher risk of hypertension [62,63]. Given the numerous and complex factors involved in the development of hypertension, VK may exert its effects through multiple pathways. Endothelial dysfunction, considered the initial event in the pathogenesis of hypertension, is characterized by reduced production of nitric oxide and vasodilatory prostanoids, which regulate vascular tone and, consequently, BP [64,65]. An in vivo study in rats observed a reduction in BP following VK1 supplementation, an effect that was shown to be mediated by activation of the nitric oxide pathway and the release of vasodilatory prostanoids [66].

Inflammatory processes (such as immune cell infiltration, cytokine release, and oxidative stress) contribute to increased BP by promoting vascular remodeling and impairing renal function [67]. Both VK1 and VK2 (MK-4) have demonstrated anti-inflammatory properties, as they can inhibit NF-κB activation and reduce the expression of cytokines involved in the pathophysiology of hypertension, including MCP-1, TNF-α, IL-6, and IL-1β, in THP-1 cells exposed to lipopolysaccharide and high glucose, as well as in T2D mouse models [67,68,69].

Another important factor is arterial calcification, particularly medial vascular calcification, which is associated with arterial stiffness and is a major contributor to systolic hypertension [70]. A study in rat vascular smooth muscle cells showed that VK2 (MK-4) inhibits calcium deposition, suppresses the expression of Runx2, a transcription factor involved in osteogenesis, and modulates the BMP-2/SMAD signaling pathway [71]. Furthermore, VK2 supplementation in animal models improved arterial calcification, and in postmenopausal women, VK1 enhanced elasticity and distensibility of the common carotid artery [72,73]. These findings suggest that VK is a promising candidate for BP reduction; however, evidence from human studies remains limited.

Following the intervention, no significant effects were observed in glucose or insulin concentrations, nor in the HOMA-IR index. VK supplementation in individuals with T2D has shown beneficial effects on fasting glucose, glycated hemoglobin, and HOMA-IR [33,34,35,36]. Similarly, individuals in the highest quartile of VK intake have been found to have a 21% lower risk of developing T2D compared to those in the lowest quartile, suggesting that VK supplementation may help improve glycemic control and reduce T2D incidence [34]. However, clinical trials evaluating VK interventions in healthy adults have not reported positive results in glucose metabolism-related variables [34,57,74,75].

Evidence suggests that VK may act through various mechanisms to improve glucose metabolism-related variables. On one hand, by reducing proinflammatory cytokines involved in IR, such as TNF-α and IL-6, and by improving the lipid profile, which is considered one of the primary mechanisms of obesity-induced IR [76,77]. On the other hand, increased adiponectin levels, an anti-inflammatory molecule that enhances insulin sensitivity, and elevated insulin levels have been observed, likely due to improved pancreatic β-cell function mediated by osteocalcin [34]. Since individuals with T2D exhibit more pronounced disturbances in plasma glucose compared to healthy individuals, the hypoglycemic effect of VK may be specific to certain pathological conditions such as T2D [33,34].

Although studies in healthy individuals have not shown consistent positive results, a major limitation of clinical trials is the limited investigation of glucose tolerance [34]. Impaired fasting glucose and impaired glucose tolerance are two metabolic disorders with distinct pathophysiologies; for this reason, the effectiveness of interventions to reverse them may also vary [78]. In healthy young adults, an intervention with VK2 (MK-4) did not result in differences in fasting glucose or insulin concentrations. However, during the oral glucose tolerance test, the area under the curve of the insulin/glucose ratio between 0 and 120 min was significantly reduced [75]. In the same population, Choi et al. reported an improvement in the insulin sensitivity index following four weeks of VK2 (MK-4) supplementation [57].

Elevated LDL cholesterol levels are the third leading modifiable risk factor for CVD mortality, following systolic BP and dietary factors [60]. In this study, we not only observed a decrease in total cholesterol of 10.64 mg/dL with a medium effect size, but also a reduction of 6.12 mg/dL in LDL cholesterol with a large effect size [55]. VK2 intake has been inversely associated with serum LDL cholesterol levels in adults [79]. Similarly, a clinical trial in children with obesity who received VK2 (MK-7) supplementation reported reductions in both total and LDL cholesterol levels [40].

That same study reported a decrease in triglyceride levels; however, this effect was not observed in the supplementation group in our study. It is important to note that the placebo group presented significantly higher baseline triglyceride concentrations, which may have influenced the observed outcomes. Although changes were analyzed using models adjusted for baseline values, the impact of between-group differences remains evident, consistent with the pattern previously observed for BP. Notably, the supplementation group started the study with triglyceride levels below the population median reported for young Mexican adults (~139.6 mg/dL), whereas the placebo group exhibited higher baseline values [80]. This difference is relevant, as it may have amplified a regression-to-the-mean effect during the study: triglyceride levels decreased in the placebo group and increased in the supplementation group, with both groups tending toward values closer to the population median [81].

The hypolipidemic effect of VK appears to be mediated by the carboxylated Gas6. Following VK1 intervention in both in vitro and in vivo models, a significant increase in AMPK phosphorylation was observed, leading to decreased mRNA and protein expression of SREBP1 and FAS, key molecules in the de novo lipogenesis signaling pathway. As well as increased expression of PPARα, CPT1A, and UCP2, which are involved in the fatty acid oxidation pathway [82].

VK supplementation has shown mixed effects on the lipid profile across different adult populations. Studies in postmenopausal women, as well as patients with vascular disease or rheumatoid arthritis, reported no significant changes [37,83,84,85]. In contrast, a clinical trial in participants with T2D found improvements in total cholesterol levels [33,39,86]. Similarly, a study in individuals with chronic kidney disease reported results consistent with those observed in this investigation [41]. These findings suggest that the effects of VK on the lipid profile may depend on the characteristics of the studied population, highlighting the need for further research to fully understand its potential therapeutic implications.

Although the reductions observed in total cholesterol and LDL cholesterol were modest, their clinical relevance should be interpreted within the context of the study population. Participants were young adults without established dyslipidemia, and baseline lipid levels in our cohort were substantially lower than those typically observed in individuals with hyperlipidemia. Therefore, conventional lipid-lowering thresholds, which are primarily defined for therapeutic interventions in high-risk populations, may not be directly applicable in this setting. From a preventive perspective, even small reductions in LDL cholesterol may be meaningful, as cumulative exposure to lower lipid levels over time has been associated with reduced cardiovascular risk [87]. In this sense, the observed changes may reflect early metabolic modulation rather than therapeutic correction, highlighting the potential role of VK in primordial prevention.

Diet constitutes a key determinant of cardiometabolic health; therefore, even transient changes in dietary intake may influence the regulation of relevant biomarkers [88,89]. In the present study, no significant changes were observed in most nutrients; however, VK2 intake increased in the placebo group following the intervention. Despite this finding, total VK intake remained stable between groups throughout the study period.

Notably, the assessment of dietary VK2 intake relied partially on external databases and published values for MK-5 to MK-11 isoforms, which are limited by the lack of comprehensive data on their content in foods. Consequently, intake estimates may be imprecise. Further efforts to generate more complete and accurate food composition data, both in Mexico and globally, are needed to enable a more robust evaluation of VK2 intake. Until such data becomes available, these findings should be interpreted with caution.

High-dose VK2 supplementation (45–90 mg/day of MK-4) has been reported to cause gastrointestinal symptoms in some studies; however, doses ranging from 50 µg/day to 600 µg/day are generally considered safe [90]. In this study, one participant in the supplementation group reported gastrointestinal symptoms shortly after initiating the intervention, which were not associated with any other concurrent events.

Some limitations of this study should be acknowledged and addressed in future research. Participants received a dose of VK2 (MK-4) of 100 μg/day, which was selected based on prior evidence used for our sample size calculation. Notably, this dose, close to the adequate intake level for VK, has previously been shown to increase cOC, our primary outcome, and to improve cardiometabolic risk factors in Mexican adults, a population with characteristics comparable to those of our study participants [36,91].

However, in the present study, these effects were not observed to the same extent, suggesting that higher doses may be required to induce measurable changes in cOC in healthy populations, consistent with previous clinical trials [92,93]. In this context, VK exerts its effects through multiple vitamin K-dependent pathways, which may differ in their sensitivity to supplementation dose and physiological roles. Therefore, the selected dose may have been sufficient to influence certain pathways without producing detectable changes in cOC levels.

Based on these considerations, future studies evaluating the effects of VK on carboxylated osteocalcin in healthy adults should consider using higher doses of VK2 (MK-4), in the range of 600 to 1000 μg/day.

In this study, obesity was defined solely based on BMI. It is currently recommended to complement this assessment with an additional parameter that allows a more direct measurement of body fat mass, such as waist circumference [94]. Although waist circumference was measured in this study, it was not used as an inclusion criterion. Therefore, it is suggested that future studies include this parameter among their selection criteria.

Further studies are needed to better characterize changes in the lipid profile. The hypolipidemic effect observed in this study may be mediated by carboxylated Gas6; however, serum levels of this form were not assessed. Therefore, quantification of both total and carboxylated Gas6 is recommended to evaluate changes in their ratio. Measuring carboxylated Gas6 in future studies will be essential to clarify its role in the effects of VK2 supplementation on the lipid profile. In addition, we were unable to adequately assess serum triglycerides concentration and BP because the placebo group started the intervention with higher baseline values for these variables. Future studies could include triglyceride concentrations and BP to better determine the impact of VK2 supplementation on these parameters.

Oxidized LDL cholesterol is a modified form of LDL that plays a crucial role in the development of atherosclerosis. It is formed when LDL cholesterol undergoes oxidative modifications, including lipid peroxidation and esterification, within the arterial wall [95,96]. This form of cholesterol contributes to atherogenesis through several mechanisms, such as promoting foam cell formation, recruiting monocytes to the intima, and damaging endothelial cells [95]. Since VK possesses properties beyond those evaluated in this study, such as antioxidant activity, it is plausible that, in addition to its demonstrated ability to reduce LDL cholesterol levels, it may also reduce oxidized LDL cholesterol concentrations [97]. Therefore, the combined evaluation of both parameters is proposed for future studies.

Since the sample size calculation was based on our primary outcome, carboxylated osteocalcin, the effects identified in secondary outcomes should be interpreted with caution and considered exploratory. Therefore, we recommend using the results of this study to design future clinical trials with primary outcomes focused on key findings, such as total cholesterol, LDL, or blood pressure. This would allow for a proper sample size calculation and more robust confirmation of the observed effects within the VK2 intervention.

Future studies should consider increasing the sample size, as well as extending both the duration of the intervention and the post-supplementation follow-up period. This would allow for the evaluation of the effects of VK in a larger population, a more comprehensive assessment of lipid profile changes over longer timeframes, and, importantly, the determination of whether these effects are sustained, attenuated, or reversed after discontinuation of supplementation. Such an approach would provide a deeper understanding of the biological and clinical relevance of the observed findings.

Finally, several mechanistic explanations mentioned above are based on findings from previous studies; however, they were not directly assessed in the present study. Therefore, future research should include the evaluation of cardiometabolic variables alongside the factors that may underlie the proposed mechanisms of action, in order to comprehensively address the effects of the intervention.

The present study comprehensively evaluated the effect of VK2 (MK-4) supplementation on cardiometabolic risk parameters. It was conducted in a vulnerable population, namely adults with overweight or obesity, a condition widely recognized as a risk factor for CVD, T2D, cancer, and other chronic conditions [98,99]. In addition, both VK1 and VK2 intake were assessed throughout the clinical trial.

## 5. Conclusions

In this randomized controlled trial, VK2 (MK-4) supplementation resulted in significant reductions in total and LDL cholesterol levels, while no significant changes were observed in vitamin K-dependent proteins. Although further research is warranted, these findings suggest that VK2 supplementation may represent a safe and potentially beneficial adjunct to nutritional strategies aimed at early cardiometabolic risk modulation.

## Figures and Tables

**Table 1:** Baseline characteristics of participants.

Variable	Total (*n* = 51)	Placebo Group (*n* = 27)	Supplementation Group (*n* = 24)	*p*
Age (years) Women (*n*, %)	26.96 ± 4.08	27.59 ± 4.55	26.25 ± 3.45	0.246 ^a^
	21 (41.2)	10 (37.0)	11 (45.8)	0.524 ^b^
Body composition				
BMI (kg/m^2^)	29.96 [27.80–33.04]	30.07 [27.85–31.94]	29.94 [27.54–33.71]	0.792 ^c^
Waist circumference (cm)	95.87 ± 10.21	97.27 ± 10.79	94.29 ± 9.48	0.304 ^a^
Fat mass (%)	34.09 ± 7.36	34.60 ± 7.89	33.52 ± 6.84	0.608 ^a^
Fat-free mass (kg)	57.94 ± 9.81	57.98 ± 8.39	57.90 ± 11.38	0.843 ^a^
Blood Pressure				
Systolic BP (mm Hg)	119.59 ± 13.94	122.37 ± 14.03	116.46 ± 13.44	0.132 ^a^
Diastolic BP (mm Hg)	78.35 ± 10.85	81.93 ± 10.87	74.33 ± 9.50	0.019 ^a^
Glucose homeostasis				
Glucose (mg/dL)	88.76 ± 9.28	90.37 ± 7.86	86.96 ± 10.53	0.193 ^a^
Insulin (μU/mL)	18.58 [10.21–25.02]	20.68 [13.14–28.87]	16.38 [10.12–23.39]	0.220 ^c^
HOMA-IR	4.60 ± 2.70	5.06 ± 2.75	4.09 ± 2.61	0.179 ^a^
Blood lipid profile				
Triglycerides (mg/dL)	139.00 [89.00–180.00]	168.00 [102.00–210.00]	115.50 [79.00–153.00]	0.049 ^c^
Total cholesterol (mg/dL)	168.71 ± 38.05	172.63 ± 46.94	164.29 ± 24.87	0.426 ^a^
HDL Cholesterol (mg/dL)	37.73 ± 10.11	36.59 ± 11.61	39.00 ± 8.16	0.402 ^a^
LDL Cholesterol (mg/dL)	99.16 ± 30.34	101.00 ± 34.43	97.08 ± 25.54	0.650 ^a^
Vitamin K-dependent proteins				
ucOC (ng/mL)	1.12 [0.54–3.49]	0.95 [0.54–3.48]	2.06 [0.41–3.72]	0.533 ^c^
cOC (ng/mL)	3.88 [2.84–6.64]	3.60 [2.60–6.64]	4.13 [3.09–7.23]	0.317 ^c^
UCR	0.49 ± 0.52	0.46 ± 0.53	0.52 ± 0.51	0.619 ^a^
Gas6 (ng/mL)	19.85 ± 3.38	19.49 ± 3.56	20.24 ± 3.19	0.664 ^a^

Variables are presented as frequency (percentage), mean ± standard deviation, or median [25th–75th percentile]. BMI, body mass index; BP, blood pressure; HOMA-IR, homeostasis model assessment of insulin resistance; ucOC, undercarboxylated osteocalcin; cOC, carboxylated osteocalcin; UCR, ucOC-to-cOC ratio; Gas6, growth arrest-specific 6. Data comparisons: Results obtained by (^a^) Independent *t*-test, (^b^) Chi-square test, and (^c^) Mann–Whitney U test.

**Table 2:** Baseline lifestyle characteristics of the participants.

Variable	Total (*n* = 51)	Placebo Group (*n* = 27)	Supplementation Group (*n* = 24)	*p*
Smoking	10 (19.6)	6 (22.2)	4 (16.7)	0.731 ^a^
Energy (kcal/day)	2002.89 [1627.15–2614.52]	2002.89 [1630.28–2614.52]	1933.41 [1504.30–2604.11]	0.597 ^b^
Protein (g/day)	92.83 [75.16–136.33]	92.83 [79.80–136.33]	93.17 [69.10–141.04]	0.485 ^b^
Carbohydrates (g/day)	214.14 [166.10–284.05]	239.86 [164.00–284.94]	209.94 [170.79–260.83]	0.734 ^b^
Total fiber (g/day)	78.16 [52.99–98.48]	19.27 [13.22–30.98]	19.70 [17.30–27.46]	0.748 ^b^
Lipids (g/day)	19.29 [15.19–28.33]	81.04 [57.62–98.93]	67.33 [52.96–95.02]	0.213 ^b^
SFA (g/day)	22.82 [16.71–31.94]	25.28 [17.75–35.74]	21.57 [15.23–31.15]	0.266 ^b^
MUFA (g/day)	22.19 [14.98–30.35]	24.38 [14.58–30.35]	20.57 [14.75–32.33]	0.685 ^b^
PUFA (g/day)	14.28 [9.34–25.21]	15.20 [9.85–25.21]	13.67 [7.75–25.64]	0.450 ^b^
TFA (g/day)	0.56 [0.28–1.21]	0.56 [0.28–1.21]	0.60 [0.27–1.25]	0.741 ^b^
Cholesterol (mg/day)	449.68 [215.68–571.67]	490.68 [217.77–612.96]	413.24 [170.56–547.63]	0.258 ^b^
Sodium (mg/day)	2036.08 [1420.59–2899.16]	2077.30 [1534.88–3651.50]	2010.01 [1282.87–2510.19]	0.282 ^b^
Vitamin D (μg/day)	3.89 [2.10–8.72]	5.72 [1.30–9.36]	3.73 [2.12–8.11]	0.777 ^b^
VK1 (μg/day)	135.08 [80.96–196.61]	135.08 [90.29–188.52]	120.45 [69.86–306.36]	0.365 ^b^
VK2 (μg/day)	91.26 [55.15–186.97]	91.26 [55.15–135.59]	98.05 [56.65–254.91]	0.925 ^b^
Total VK (μg/day)	11.06 [2.55–45.41]	11.06 [2.49–73.84]	12.20 [2.83–27.47]	0.940 ^b^
Physical activity level				
Low	19 (37.2)	11 (40.7)	8 (33.3)	0.797 ^a^
Moderate	23 (45.1)	11 (40.7)	12 (50.0)	
High	9 (17.6)	5 (18.5)	4 (16.7)	

Variables are presented as frequency (percentage) or median [25th–75th percentile]. SFA, saturated fatty acids; MUFA, monounsaturated fatty acids; PUFA, polyunsaturated fatty acids; TFA, trans fatty acids; VK, vitamin K. Data comparisons: Results obtained by (^a^) Chi-square test and (^b^) Mann–Whitney U test.

**Table 3:** Changes between groups after the 12-week intervention on cardiometabolic risk factors.

Variable	Δ Placebo Group (*n* = 24)	Δ Supplementation Group (*n* = 22)	*p*
Body composition			
BMI (kg/m^2^)	0.24 (−0.03–0.53)	0.04 (−0.25–0.33)	0.319
Waist circumference (cm) *	0.66 (−0.38–1.70)	−0.51 (−1.61–0.57)	0.141
Fat mass (%)	−0.16 (−1.00–0.66)	−0.14 (−1.01–0.72)	0.996
Fat-free mass (kg)	0.44 (−0.10–0.99)	0.21 (−0.36–0.78)	0.552
Blood Pressure			
Systolic BP (mm Hg)	−0.69 (−4.25–2.87)	−5.56 (−9.29–−1.83)	0.067
Diastolic BP (mm Hg)	−4.84 (−8.96–−0.72)	−7.16 (−11.49–−2.84)	0.453
Glucose homeostasis			
Glucose (mg/dL)	1.39 (−1.74–4.52)	−1.60 (−4.88–1.66)	0.195
Insulin (μU/mL) *	4.47 (−0.91–9.86)	3.44 (−2.19–9.08)	0.534
HOMA-IR	1.08 (−0.13–2.30)	0.69 (−0.57–1.96)	0.389
Blood lipid profile			
Triglycerides (mg/dL)	−31.06 (−55.25–−6.87)	0.34 (−24.92–25.61)	0.054
Total cholesterol (mg/dL) *	3.58 (−5.59–12.76)	−10.64(−20.23–−1.04)	0.038
HDL Cholesterol (mg/dL)	−1.99 (−5.08–1.10)	−4.78 (−8.01–−1.55)	0.215
LDL Cholesterol (mg/dL) *	11.82 (3.42–20.21)	−6.12 (−14.89–2.65)	0.005
Vitamin K-dependent proteins			
ucOC (ng/mL) *	−0.19 (−0.47–0.08)	−0.04 (−0.33–0.25)	0.333
cOC (ng/mL) *	0.42 (−0.36–1.21)	−0.02 (−0.84–0.80)	0.079
UCR *	−0.08 (−0.23–0.07)	0.06 (−0.09–0.22)	0.081
Gas6 (ng/mL)	−0.31 (−1.25–0.61)	−0.44 (−1.42–0.52)	0.848

Variables are presented as estimated mean difference (95% CI). BMI, body mass index; BP, blood pressure; HOMA-IR, homeostasis model assessment of insulin resistance; ucOC, undercarboxylated osteocalcin; cOC, carboxylated osteocalcin; UCR, ucOC-to-cOC ratio; Gas6, growth arrest-specific 6. Data comparisons were performed using analysis of covariance (ANCOVA) with Bonferroni adjustment for multiple comparisons, adjusted for baseline values. * The variable was logarithmically transformed to achieve normality and then back-transformed for the presentation of results.

**Table 4:** Changes between groups after the 12-week intervention on nutrient intake.

Variable	Δ Placebo Group (*n* = 24)	Δ Supplementation Group (*n* = 22)	*p*
Energy (kcal/day)	−63.12 (−343.53–217.28)	−104.07 (−396.95–188.81)	0.840
Protein (g/day)	−2.72 (−18.91–13.46)	−4.90 (−21.82–12.00)	0.852
Carbohydrates (g/day)	−1.05 (−34.77–32.66)	−13.15 (−48.37–22.06)	0.619
Total fiber (g/day) *	−2.37 (−6.19–1.44)	−1.02 (−5.00–2.95)	0.942
Lipids (g/day) *	−9.44 (−24.17–5.28)	−2.93 (−18.33–12.45)	0.404
SFA (g/day) *	−1.85 (−7.65–3.95)	−1.69 (−7.75–4.36)	0.800
MUFA (g/day) *	−0.55 (−5.94–4.82)	−0.68 (−4.94–6.31)	0.927
PUFA (g/day)	−1.25 (−5.71–3.19)	1.70 (−2.95–6.35)	0.359
TFA (g/day) *	−0.47 (−1.14–0.19)	−0.09 (−0.79–0.60)	0.720
Cholesterol (mg/day) *	−79.74 (−174.62–15.14)	−150.37 (−249.48–−51.27)	0.180
Sodium (mg/day)	−142.94 (−300.23–586.11)	−104.67 (−567.58–358.22)	0.440
Vitamin D (μg/day)	−0.49 (−2.31–1.31)	−1.47 (−3.36–0.42)	0.460
VK1 (μg/day) *	−26.64 (−50.23–−3.05)	−28.06 (−52.73–−3.40)	0.729
VK2 (μg/day) *	125.24 (44.59–205.88)	−5.54 (−89.77–78.68)	0.041
Total VK (μg/day) *	93.05 (5.70–180.40)	−24.13 (−117.59–69.32)	0.118

Variables are presented as estimated mean difference (95% CI). SFA, saturated fatty acids; MUFA, monounsaturated fatty acids; PUFA, polyunsaturated fatty acids; TFA, trans fatty acids; VK, vitamin K. Data comparisons were performed using analysis of covariance (ANCOVA) with Bonferroni adjustment for multiple comparisons, adjusted for baseline values. * The variable was logarithmically transformed to achieve normality and then back-transformed for the presentation of results.

## Data Availability

Raw data supporting the findings of this study are available upon reasonable request from the corresponding author to ensure participant anonymity and comply with data protection regulations.

## Supplementary Materials

The following supporting information can be downloaded at: https://www.mdpi.com/article/10.3390/biomedicines14051011/s1, Table S1: Changes over time in nutrient intake in the placebo group; Table S2: Changes over time in nutrient intake in the supplementation group.

## Abbreviations

The following abbreviations are used in this manuscript:

VK2	Vitamin K2
IR	Insulin resistance
BP	Blood pressure
CVD	Cardiovascular disease
T2D	Type 2 diabetes
VK	Vitamin K
VK1	Vitamin K1
MK	Menaquinone
Gas6	Growth arrest-specific 6
CONSORT	Consolidated Standards of Reporting Trials
BMI	Body mass index
cOC	Carboxylated osteocalcin
ELISA	Enzyme-linked immunosorbent assay
HOMA-IR	Homeostasis model assessment of insulin resistance
ucOC	Undercarboxylated osteocalcin
UCR	ucOC-to-cOC ratio
FNDDS	Food and Nutrient Database for Dietary Studies
USDA	United States Department of Agriculture
IPAQ	International Physical Activity Questionnaire
ANCOVA	Analysis of covariance
SFA	Saturated fatty acids
MUFA	Monounsaturated fatty acids
PUFA	Polyunsaturated fatty acids
TFA	Trans fatty acids
