# Supplementary material for: Vitamin K2 Supplementation Reduces Cardiometabolic Risk Factors in Young Adults with Overweight and Obesity—A Randomized Placebo-Controlled Trial
Source: Biomedicines. 2026 Apr 29;14(5):1011. doi: 10.3390/biomedicines14051011 (PMC13204484; doi:10.3390/biomedicines14051011)
Supplement: Supplementary file 1 [file biomedicines-14-01011-s001.zip › biomedicines-4275844-supplementary.pdf]

Table S1. Changes over time in nutrient intake in the placebo group

Variable	Baseline (n = 27)	4 weeks (n = 24)	8 weeks (n = 24)	12 weeks (n = 24)	p
Energy (kcal/day)	2002.89 [1630.28 - 2614.52]	1924.39 [1577.95 - 2450.88]	2091.24 [1663.99 - 3057.63]	2026.48 [1468.55 - 2462.12]	0.236
Protein (g/day)	92.83 [79.80 - 136.33]	101.69 [80.72 - 123.65]	111.14 [91.27 - 141.98]	97.50 [71.65 - 126.27]	0.384
Carbohydrates (g/day)	239.86 [164.00 - 284.94]	223.77 [154.76 - 274.03]	229.32 [170.74 - 312.56]	209.74 [166.64 - 289.01]	0.432
Total fiber (g/day)	19.27 [13.22 - 30.98]	18.63 [13.97 - 25.99]	19.72 [15.37 - 28.51]	17.86 [13.44 - 25.42]	0.950
Lipids (g/day)	81.04 [57.62 - 98.93]	74.33 [57.64 - 101.43]	80.12 [58.52 - 111.81]	62.35 [44.30 - 92.28]	0.321
SFA (g/day)	25.28 [17.75 - 35.74]	24.59 [17.39 - 36.25]	26.70 [20.68 - 39.22]	22.63 [12.49 - 29.14]	0.148
MUFA (g/day)	24.38 [14.58 - 30.35]	24.93 [19.89 - 31.39]	22.29 [16.49 - 35.90]	21.99 [15.27 - 28.51]	0.175
PUFA (g/day)	15.20 [9.85 - 25.21]	16.14 [11.90 - 23.98]	13.98 [9.46 - 24.22]	11.42 [10.14 - 21.79]	0.522
TFA (g/day)	0.56 [0.28 - 1.21]	0.27 [0.11 - 0.61]	0.68 [0.15 - 1.36]	0.33 [0.06 - 0.86]	0.302
Cholesterol (mg/day)	490.68 [217.77 - 612.96]	324.80 [202.30 - 495.37]	423.96 [271.77 - 533.24]	365.95 [191.77 - 568.03]	0.278
Sodium (mg/day)	2077.30 [1534.88 - 3651.50][a]	2794.46 [1732.24 - 3756.52][b]	2837.03 [2148.07 - 3231.39][ac]	2101.78 [1571.53 - 2803.85] [abd]	0.001
Vitamin D (µg/day)	5.72 [1.30 - 9.36]	4.60 [1.47 - 10.83]	5.06 [2.96 - 9.42]	2.58 [0.97 - 7.79]	0.626
Total VK (µg/day)	135.08 [90.29 - 188.52]			164.79 [94.27 - 313.12]	0.110
VK1 (µg/day)	91.26 [55.15 -135.59]			77.54 [53.87 - 143.48]	0.753
VK2 (µg/day)	11.06 [2.49 - 73.84]	25.79 [6.23 – 64.80]	26.06 [6.26 – 99.35]	72.07 [13.65 - 187.34]	0.253

Variables are presented as median [25th–75th percentile]. SFA, saturated fatty acids; MUFA, monounsaturated fatty acids; PUFA, polyunsaturated fatty acids; TFA, trans fatty acids; VK, vitamin K. Data comparisons: Results obtained by Wilcoxon signed-rank or Friedman test. Equal letters indicate no statistically significant differences between groups, while different letters indicate statistically significant differences ($p < 0.05$).

Table S2. Changes over time in nutrient intake in the supplementation group

Variable	Baseline (n = 24)	4 weeks (n = 24)	8 weeks (n = 23)	12 weeks (n = 22)	p
Energy (kcal/day)	1933.41 [1504.30 - 2604.11]	1967.06 [1505.32 - 2367.79]	2096.38 [1451.53 - 2369.00]	2015.33 [1366.24 - 2393.42]	0.392
Protein (g/day)	93.17 [69.10 - 141.04]	78.95 [59.75 - 101.64]	88.45 [72.14 - 123.46]	92.67 [50.25 - 144.34]	0.806
Carbohydrates (g/day)	209.94 [170.79 - 260.83]	218.25 [153.85 - 296.86]	201.23 [142.62 - 246.48]	201.22 [154.59 - 281.20]	0.714
Total fiber (g/day)	19.70 [17.30 - 27.46]	19.28 [13.55 - 24.96]	20.07 [14.46 - 25.16]	18.94 [13.77 - 26.14]	0.445
Lipids (g/day)	67.33 [52.96 - 95.02]	66.36 [53.29 - 100.51]	71.41 [51.12 - 89.02]	76.68 [48.35 - 94.69]	0.975
SFA (g/day)	21.57 [15.23 - 31.15]	19.57 [17.87 - 28.52]	21.51 [17.53 - 27.36]	23.49 [12.47 - 30.86]	0.921
MUFA (g/day)	20.57 [14.75 - 32.33]	19.43 [14.64 - 28.40]	17.49 [15.27 - 27.86]	18.74 [14.14 - 36.31]	0.944
PUFA (g/day)	13.67 [7.75 - 25.64]	13.59 [11.20 - 21.12]	15.49 [7.38 - 24.98]	16.18 [9.42 - 28.36]	0.806
TFA (g/day)	0.60 [0.27 - 1.25]	0.44 [0.08 - 1.21]	0.44 [0.29 - 1.30]	0.32 [0.10 -1.00]	0.281
Cholesterol (mg/day)	413.24 [170.56 - 547.63]	300.77 [152.52 - 477.70]	267.34 [138.55 - 545.05]	255.39 [95.93 - 469.00]	0.484
Sodium (mg/day)	2010.01 [1282.87 - 2510.19]	2490.06 [1374.02 - 2947.58]	2589.35 [1649.80 - 3301.48]	1967.50 [995.39 - 3385.10]	0.288
Vitamin D (µg/day)	3.73 [2.12 - 8.11]	3.08 [1.62 - 4.88]	2.87 [0.96 - 5.55]	2.74 [1.14 - 5.14]	0.740
Total VK (µg/day)	120.45 [69.86 - 306.36]			144.20 [72.59 - 198.59]	0.122
VK1 (µg/day)	98.05 [56.65 - 254.91]			92.65 [60.02 - 171.78]	0.039
VK2 (µg/day)	12.20 [2.83 - 27.47][a]	20.70 [6.70 - 59.44][ac]	59.53 [21.20 - 61.89][bc]	18.34 [1.16 - 55.40][ac]	0.036

Variables are presented as median [25th–75th percentile]. SFA, saturated fatty acids; MUFA, monounsaturated fatty acids; PUFA, polyunsaturated fatty acids; TFA, trans fatty acids; VK, vitamin K. Data comparisons: Results obtained by Wilcoxon signed-rank t or Friedman test. Equal letters indicate no statistically significant differences between groups, while different letters indicate statistically significant differences ($p < 0.05$).
